# The Mechanism Study of Moxa Combustion Products on Regulating Vascular Endothelial Function in Atherosclerotic Mice

**DOI:** 10.1155/2022/1303978

**Published:** 2022-10-03

**Authors:** Huang Yueping, Yao Qin, OuYang Xiali, Lin Yao, Liu Yajie, Huang Chang, He Rui, Hui Xin, Wang Hao, Zhang Rui, Liu Jinyi, Zhao Baixiao

**Affiliations:** ^1^School of Acupuncture-Moxibustion and Tuina, Beijing University of Chinese Medicine, Beijing 102488, China; ^2^School of Life Science, Beijing University of Chinese Medicine, Beijing 102488, China; ^3^Beijing Aerospace General Hospital, Beijing 100076, China; ^4^Chinese PLA General Hospital, Beijing 100039, China; ^5^School of Acupuncture-Moxibustion and Tuina, Shaanxi University of Chinese Medicine, Xianyang 712000, China; ^6^Dongzhimen Hospital Affiliated to Beijing University of Chinese Medicine, Beijing 100700, China

## Abstract

**Objective:**

To evaluate the anti-atherogenic effect of moxa combustion products (MCPs) and whether it is mediated through improving the vascular endothelial function in ApoE^−/−^ mice.

**Methods:**

A total of 60 male ApoE^−/−^ mice were randomly divided into the moxa smoke (MS) group, filtered moxa smoke (FMS) group, moxa floss volatile (MFV) group, essential oil of *Artemisia argyi* (EOAA) group, and model group (*n* = 12/group), while 12 male C57BL/6 mice were used as the control group. The six groups were intervened for 20 min/day, 6 days/week. After 14 weeks of intervention, the mice were euthanized and their blood lipids were measured. The aortic roots and thoracic aortas were collected for haematoxylin and eosin (HE) or Oil Red O staining, respectively. The contents of AMPK, PI3K, Akt, and eNOS mRNA in the thoracic aortas were examined by RT-qPCR.

**Results:**

The MS group and FMS group showed significantly lower plaque area percentage in the aortic roots and thoracic aortas and higher contents of AMPK-mRNA and eNOS-mRNA in the thoracic aortas compared with the model group.

**Conclusion:**

MS and FMS equally suppressed the progression of atherosclerotic lesions in ApoE^−/−^ mice. It was suggested that the particulate matter in MS may not be the key components of moxibustion.

## 1. Introduction

Atherosclerosis (AS) is a kind of chronic inflammatory disease that occurs in the large or medium arteries, where lipids and inflammatory cells gather and form fibrous plaques, resulting in vascular lumen stenosis gradually [2, 3]. It is generally known that AS is the primary cause of cardiovascular disease, which has become the main inducement of death and disability worldwide [4]. AS can be treated with statins and other hypolipidemic drugs, which not only reduce the levels of LDL-C and TC but also induce many adverse effects. The endothelial cells (ECs) are the key components in the vascular endothelium, which play an important role in regulating vascular function mainly by regulating vasoactive component production and participating in the physiological and pathological process such as vascular tension regulation, coagulation and fibrinolysis, inflammatory reaction, and neovascularization [5]. Nitric oxide (NO) and endothelin-1 (ET-1) are antagonistic active substances synthesized by ECs, which induce vasodilation or contraction for the vascular endothelium. The imbalance of NO and ET-1 in plasma is one of the main reasons of EC dysfunction [6]. It is characterized by decreasing in the level of NO or its biological activity [7, 8] and increasing in the level of ET-1 [9]. It is worth noting that endothelial nitric oxide synthase (eNOS) is a key enzyme for the synthesis of NO, and its activation can promote the secretion of NO by ECs [10]. The AMPK could activate eNOS and promote NO production by activating the Akt-eNOS pathway or PI3K/Akt/eNOS pathway [11]. Clinical and epidemiological studies have identified a strong relationship between endothelial function and cardiovascular disease risk [12]. The vascular endothelial dysfunction (VED) is characterized by impairment in endothelial-dependent vasodilation, which participates in the whole process of AS. Thus, the therapeutic strategies designed to improve VED will be considered as the effective method to anti-AS.

Artemisinin is the medicine for treating malaria. *Artemisia argyi*, like *Artemisia annua*, belongs to the genus of composite and is the main material of moxibustion [13]. The clinical application of moxibustion and MS was recorded in “Handbook of Prescription for Emergency.” The original text was “in order to cut off the spread of pestilence, the moxa floss can be ignited and placed around the patient's bed to eliminate the virus infection.” Since the outbreak of the COVID-19 in 2020, MS has been used to sterilize the air in China and is also reported to have anti-inflammatory effect to regulate autonomic functions [14–17]. Besides, animal experiments on AS showed that the moxibustion or MS may treat cardiovascular diseases by improving the lipid metabolism, affecting the antioxidant capacity and reducing the oxidative stress damage [18–20].

Moxibustion is composed of the words “mogusa” (burning the herb (the moxa floss was commonly used)) and “combustion” (burning) [17]. Therefore, it is inevitable to produce burning substances in the process of moxibustion, which are considered as the MCPs. The MCPs mainly were divided into two kinds of components: light components, which were easy to volatilize, and heavy components, which were hard to volatilize. The light components were produced by just heating but not burning *Artemisia argyi* or moxa floss. The heavy components were produced by burning *Artemisia argyi* or moxa floss [21].

The AS in ApoE^−/−^ mice could be prevented or treated by the intervention of moxibustion or MCPs, improving the lipid metabolism, reducing the inflammatory response, and so on [19, 20]. However, the mechanism of MCP and its related components affecting the vascular endothelial function and improving AS was still unclear. Thus, we were going to observe the effect of the MCPs including MS, FMS, MFV, and EOAA for anti-AS to reveal the effective link between the moxibustion and mechanism.

## 2. Methods

### 2.1. Experimental Animals

A total of 60 male ApoE^−/−^ mice (8 weeks old, 20 ± 5 g) and 12 male C57BL/6 mice of the same background (8 weeks old, 20 ± 5 g) were purchased from Beijing Vital River Laboratory Animal Technology Co. Ltd. (SCXK (Jing) 2018-0006). For a week preceding the experiment, the mice were acclimatized to the 12-hour light/dark cycle in the environment with the temperature of 22 ± 2°C and the humidity of 50%∼60%. The ApoE^−/−^ mice were fed high-fat, cholesterol-rich/atherogenic diet (containing 15% fat, 2% cholesterol, and 0.05% cholic acid), and the C57BL/6 mice were fed common food. This experiment was approved by the Animal Ethics Committee of Beijing University of Chinese Medicine Laboratory Animal Center and conducted in accordance with the National Institutes of Health's Guide for the Care and Use of Laboratory Animals.

### 2.2. Experiment Design

A total of 60 male ApoE^−/−^ mice were randomly assigned (i.e., by a random number table (*n* = 12/group)). These groups included the MS group, FMS group, MFV group, EOAA group, and model group. In addition, 12 male C57BL/6 mice served as the control group. Animals were exposed in an automatic dynamic exposure device, which can automatically control the parameters of oxygen, humidity, temperature, and pressure. Before the intervention, the basic parameters of the device were set as follows: the oxygen was 26%, the humidity was 45%∼63%, the temperature was 20°C∼22°C, and the pressure was −70 kPa. Then, the shading rate of device was set at 2% to keep the concentration of MCPs steadily. In addition, the moxa floss and essential oil of Artemisia argyi used for intervention in the experiment were provided by the Hubei Li Shi Zhen Pharmaceutical Group Co. Ltd.

Mice in the MS group were placed in the device, and then MS was produced by burning 1.5 g moxa floss and importing the device to intervene mice. Mice in the FMS group were placed in the device where the Whatman Cambridge filters were installed at the entrance to intercept particulate matter in MS, and FMS was produced by burning 1.5 g moxa floss similarly. Mice in the MFV group were placed in the device, then 1.5 g moxa floss was evenly spread in the glass tube, the glass tube circulator was started to heat the glass tube at 150°C and moved with constant speed, and then the MFV was imported the device to intervene mice. Mice in the EOAA group were placed in the device, and 3.75 *μ*L of EOAA (equivalent to 1.5 g volatile oil of moxa floss) mixed with 16 mL distilled water was atomized and imported to the exposure device, and the atomization speed was set at 0.8 mL/min. The model and control groups were placed in the fixator just as the above groups but were not intervened. Mice intervened in the device lasted for 20 min/day, 6 days/week, and for 14 weeks.

### 2.3. Biological Sample Collection and Measurement

Twenty-four hours after the last treatment in the experiment, all mice were weighed and then anaesthetized using the intraperitoneal injection of pentobarbital (1%) at 50 mg/kg. Then, 1∼1.5 mL blood samples were collected from the common ophthalmic artery. After centrifugation for 15 min (4°C, 1500 rpm), the serum was stored at −20°C. The contents of TC, TG, HDL-C, LDL-C, and NO were measured using the automatic biochemical analyzer. The contents of ox-LDL, ApoA-I, ET-1, TXB2, and 6-keto-PGF1*α* were measured using the enzyme-linked immunosorbent assay. The cervical dislocation was conducted shortly to ensure painless and ethical death of the mice.

### 2.4. Staining of the Aortic Roots and Thoracic Aortas

After blood collection, the mice were perfused with phosphate buffer saline (0.1 mol/L), the aortic roots and thoracic aortas were dissected and fixed overnight in 4% paraformaldehyde, and then the aortic roots were paraffin-embedded and sectioned at 5 *µ*m thickness and stained with HE staining to visualize the lesions and plaque areas. The thoracic aortas were dehydrated with 20% and 30% sucrose, embedded in Leica compound, frozen immediately in liquid nitrogen, and then stored in the fridge at −80°C. Each thoracic aorta was sectioned (6 *µ*m) using the freezing microtome (Leica CM1850) and stained with Oil Red O to visualize the lipid deposition.

### 2.5. Real-Time Quantitative Polymerase Chain Reaction (RT-qPCR)

The contents of AMPK, PI3K, Akt, and eNOS mRNA in the thoracic aortas were analyzed by RT-qPCR. Total RNA was extracted with Trizol reagent according to the manufacturer's instructions from the full-length aortas. All PCR primers are listed in Table 1. A reverse transcription kit was used for reverse transcription reaction to synthesize cDNA, and RT-qPCR was performed to determine the content of AMPK, PI3K, Akt, and eNOS mRNA with the SYBR PCR mixture. Each sample was analyzed in triplicate and normalized to GAPDH. RT-qPCR conditions were 95°C for 2 min, followed by 40 cycles of 95°C for 15 s, 60°C for 45 s, 60°C for 60 s, and 95°C for 15 s.

### 2.6. Statistical Analysis

The SPSS 20.0 software was used to analyze the data. All data were tested for the normality and homogeneity of variance. The data which accorded with normality and homogeneity of variance were presented as mean ± standard deviation (SD) and analyzed by one-way ANOVA. When there were significant differences between groups, multiple comparisons between groups were made by the least significant difference (LSD) test. *P* < 0.05 was considered as significant difference.

## 3. Results

### 3.1. Changes in the Weight of Mice in Each Group

**Figure 1:**
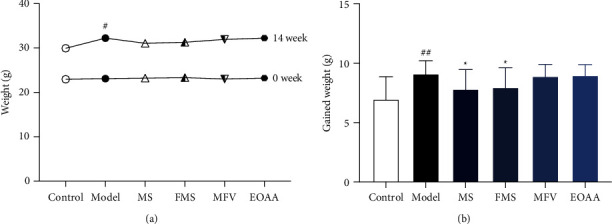
Changes in weight of ApoE^−/−^ mice. (a) The weight of each group was measured at 0 weeks and 14th week. (b) The mice gained weight from the beginning to 14 weeks. Compared with the control group, ^#^*P* < 0.05, ^##^*P* < 0.01; compared with the model group, ^*∗*^*P* < 0.05, ^*∗∗*^*P* < 0.01 (mean ± SD, *n* = 12).

The weight is the basic index of hyperlipoidemia. As shown in Figures 1(a) and 1(b), the mice's gained weight in the model group was significantly higher than that in the control group (*P* < 0.01). The mice's gained weight in the MS and FMS groups was significantly lower than that in the model group (*P* < 0.05).

### 3.2. The Contents and Comparison of TC and TG in Each Group

The content of TC in the model group was significantly higher than that in the control group (*P* < 0.01). The contents of TC in the MS group, FMS group, and MFV group were significantly lower than those in the model group (*P* < 0.01). The content of TC in the EOAA group was lower than that in the model group, but there was no significant difference (*P* > 0.05). The contents of TC among the MCP groups had significant difference (*P* < 0.05).

**Figure 2:**
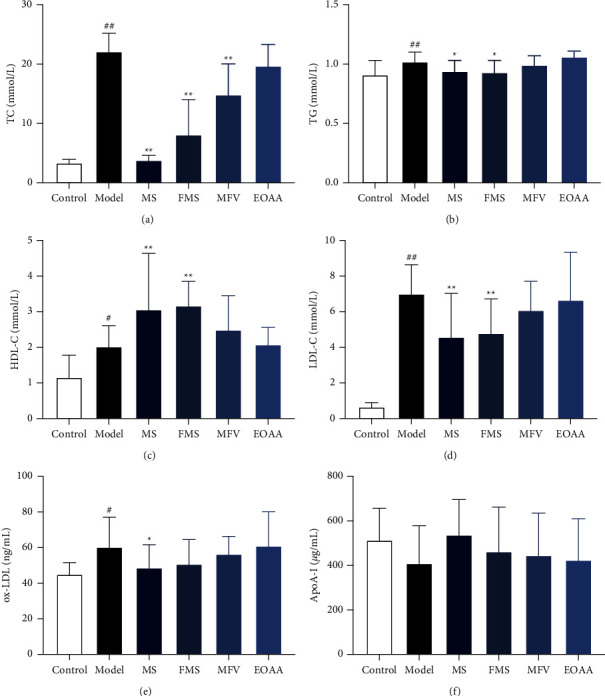
The contents of TC, TG, HDL-C, LDL-C, ox-LDL, and ApoA-I. (a) TC. (b) TG. (c) HDL-C. (d) LDL-C. (e) Ox-LDL. (f) ApoA-I. Compared with the control group, ^#^*P* < 0.05, ^##^*P* < 0.01; compared with the model group, ^*∗*^*P* < 0.05, ^*∗∗*^*P* < 0.01 (mean ± SD, *n* = 12). TC, total cholesterol; TG, triglyceride; HDL-C, high-density lipoprotein; LDL-C, low-density lipoprotein; ox-LDL, oxidized low-density lipoprotein; ApoA-I, apolipoprotein A-I.

The content of TG in the model group was significantly higher than that in the control group (*P* < 0.01). The contents of TG in the MS group and FMS group were significantly lower than those in the model group (*P* < 0.05). The contents of TG in the MFV group and EOAA group were lower than those in the model group, but there was no significant difference (*P* > 0.05). Comparing the MCP groups, the contents of TG in the MS group and FMS group were significantly lower than those in the EOAA group (*P* < 0.05), and there was no significant difference among other groups (*P* > 0.05). These results are shown in Figures 2(a) and 2(b).

### 3.3. The Contents and Comparison of HDL-C and LDL-C in Each Group

The content of HDL-C in the model group was significantly higher than that in the control group (*P* < 0.05). The contents of HDL-C in the MS group and FMS group were significantly higher than those in the model group (*P* < 0.01). The contents of HDL-C in the MFV group and EOAA group were higher than those in the model group, but there was no significant difference (*P* > 0.05). Among the MCP groups, the contents of HDL-C in the MS group and FMS group were higher than those in the EOAA group (*P* < 0.05), and there was no significant difference among other groups (*P* > 0.05).

The content of LDL-C in the model group was significantly higher than that in the control group (*P* < 0.01). The contents of LDL-C in the MS group and FMS group were significantly lower than those in the model group (*P* < 0.01). The contents of LDL-C in the MFV group and EOAA group were lower than those in the model group, but there was no significant difference (*P* > 0.05). Among the MCP groups, the content of LDL-C in the MS group was lower than that in the MFV group and EOAA group (*P* < 0.05), and there was no significant difference among other groups (*P* > 0.05). These results are shown in Figures 2(c) and 2(d).

### 3.4. The Contents and Comparison of Ox-LDL and ApoA-I in Each Group

The content of ox-LDL in the model group was significantly higher than that in the control group (*P* < 0.05). The content of ox-LDL in the MS group was significantly lower than that in the model group (*P* < 0.05). The contents of ox-LDL among the FMS group, MFV group, and EOAA group were lower than those in the model group, but there was no significant difference (*P* > 0.05). Among the MCP groups, the content of ox-LDL in the MS group was lower than that in the EOAA group (*P* < 0.05), and there was no significant difference among other groups (*P* > 0.05).

Tested by ANOVA, the contents of ApoA-I among the groups showed no significant difference (*P* > 0.05). These results are shown in Figures 2(e) and 2(f).

### 3.5. The Contents and Comparison of NO and ET-1 in Each Group

The content of NO in the model group was significantly lower than that in the control group (*P* < 0.01). The contents of NO in the MS group, FMS group, and MFV group were significantly higher than those in the model group (*P* < 0.01, *P* < 0.05, *P* < 0.05). The content of NO in the EOAA group was higher than that in the model group, but there was no significant difference (*P* > 0.05). Among the MCP groups, the content of NO in the MS group was higher than that in the EOAA group (*P* > 0.05), and there was no significant difference among other groups (*P* > 0.05).

The content of ET-1 in the model group was significantly higher than that in the control group (*P* < 0.01). The contents of ET-1 in the MS group, FMS group, and MFV group were significantly lower than those in the model group (*P* < 0.01). The content of ET-1 in the EOAA group was lower than that in the model group, but there was no significant difference (*P* > 0.05). Among the MCP groups, the contents of ET-1 in the FMS group and MFV group were lower than those in the EOAA group (*P* < 0.05), and there was no significant difference among other groups (*P* > 0.05).

**Figure 3:**
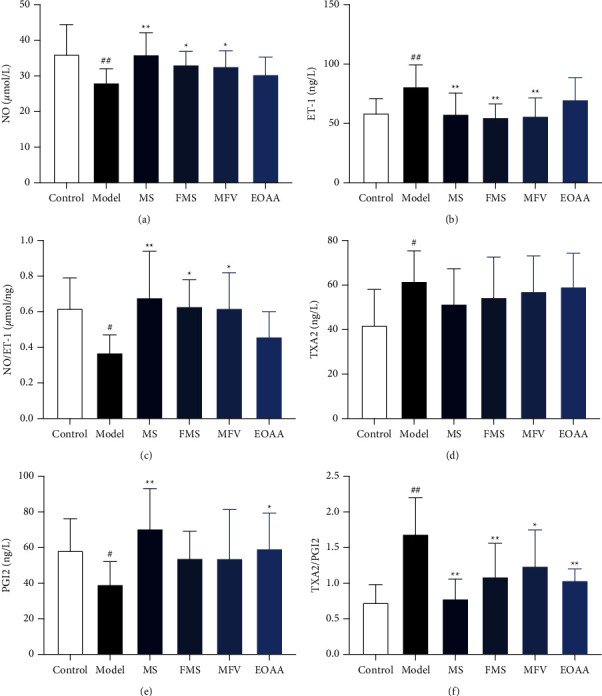
The contents of NO, ET-1, TXA2, and PGI2. The ratio of NO/ET-1 or TXA2/PGI2. (a) NO. (b) ET-1. (c) Ratio of NO/ET-1. (d) TXA2. (e) PGI2. (f) Ratio of TXA2/PGI2. Compared with the control group, ^#^*P* < 0.05, ##*P* < 0.01; compared with the model group, ^*∗*^*P* < 0.05, ^*∗∗*^*P* < 0.01 (mean ± SD, *n* = 12). NO, nitric oxide; ET-1, endothelin-1; TXA2, thromboxane A2; PGI2, prostacyclin.

The content of NO/ET-1 in the model group was significantly lower than that in the control group (*P* < 0.05). The contents of NO/ET-1 in the MS group, FMS group, and MFV group were significantly higher than those in the model group (*P* < 0.01, *P* < 0.05, *P* < 0.05). The content of NO/ET-1 in the EOAA group was higher than that in the model group, but there was no significant difference (*P* > 0.05). Among the MCP groups, the contents of NO/ET-1 in the MS group, FMS group, and MFV group were higher than those in the EOAA group (*P* < 0.05), and there was no significant difference among other groups (*P* > 0.05). These results are shown in Figures 3(a)–3(c).

### 3.6. The Contents and Comparison of TXA2 and PGI2 in Each Group

Since TXA2 and PGI2 are both unstable and easily degraded, this experiment measured the metabolites TXB2 and 6-keto-PGF1*α* to indirectly reflect the contents of TXA2 and PGI2.

The content of TXA2 in the model group was significantly higher than that in the control group (*P* < 0.05). There was no significant difference in the content of TXA2 among the model group and the MCP groups (*P* > 0.05). The contents of TXA2 among the MCP groups showed no significant difference (*P* > 0.05).

The content of PGI2 in the model group was significantly lower than that in the control group (*P* < 0.05). The contents of PGI2 in the MS group and EOAA group were significantly higher than those in the model group (*P* < 0.01, *P* < 0.05). The contents of PGI2 in the FMS group and MFV group were higher than those in the model group, but there was no significant difference (*P* > 0.05). Among the MCP groups, the content of PGI2 in the MS group was higher than that in the FMS group and MFV group (*P* < 0.05), and there was no significant difference among other groups (*P* > 0.05).

The content of TXA2/PGI2 in the model group was significantly higher than that in the control group (*P* < 0.01). The contents of TXA2/PGI2 in the MCP groups were significantly lower than those in the model group (*P* < 0.01, *P* < 0.01, *P* < 0.05, *P* < 0.01). Among the MCP groups, the content of TXA2/PGI2 in the MS group was lower than that in the MFV group (*P* < 0.05), and there was no significant difference among other groups (*P* > 0.05). These results are shown in Figures 3(d)–3(f).

### 3.7. Pathological Morphology of the Aortic Roots and Thoracic Aortas

**Figure 4:**
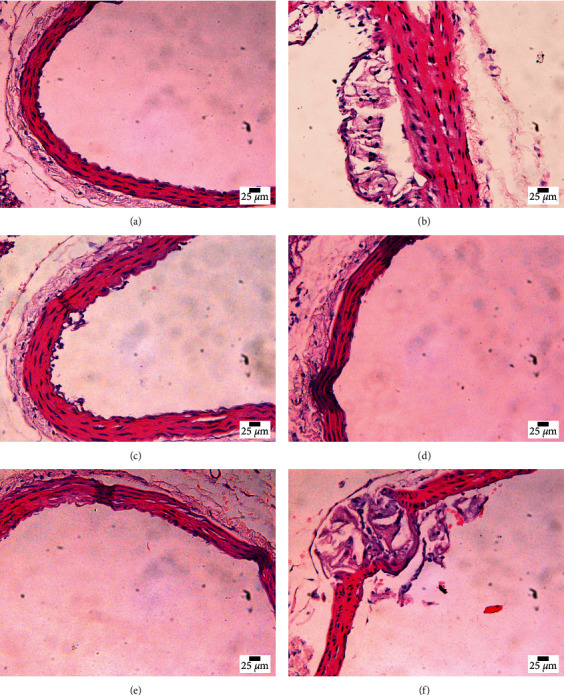
Representative images of HE staining of the aortic root in each group. (a) The control group. (b) The model group. (c) The MS group. (d) The FMS group. (e) The MFV group. (f) The EOAA group.

**Figure 5:**
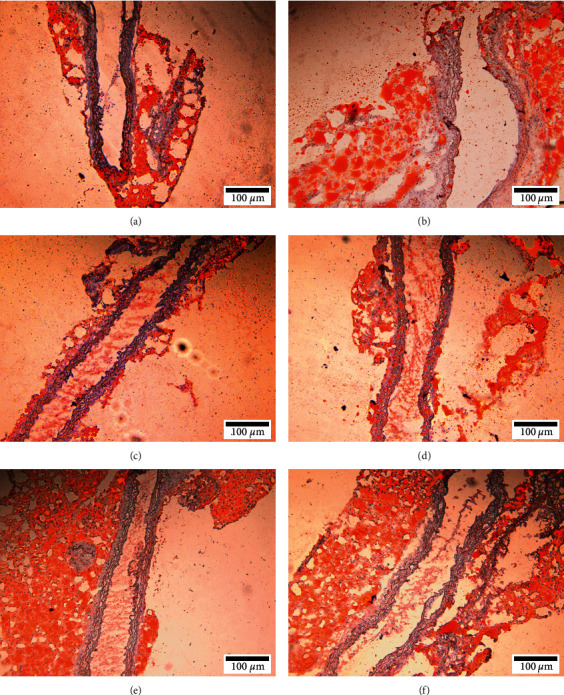
Representative images of Oil Red O staining of aortic trunk in each group. (a) The control group. (b) The model group. (c) The MS group. (d) The FMS group. (e) The MFV group. (f) The EOAA group.

The histopathological changes such as gross morphology, vascular structure, plaque structure, and lipid infiltration of the aortic roots and thoracic aortas observed in cross or longitudinal section, respectively, are shown in Figures 4(a)–4(f) and Figures 5(a)–5(f).

#### 3.7.1. The Control Group

The three-layer structures of the vascular wall were clearly visible, the vascular ECs were intact, the elastic fibers were visible, and the smooth muscles of the vascular media were arranged in order. Other abnormalities and obvious lipid infiltration were not found.

#### 3.7.2. The Model Group

The inner wall of the vascular was not smooth with the exfoliated ECs, and the elastic fibers were broken accompanied by the formation of atherosclerotic plaques and obvious stenosis of the whole vascular lumen, and there were more fat droplets in the plaques.

#### 3.7.3. The MS Group, FMS Group, and MFV Group

The three-layer structures of the vascular wall were clearly visible, few elastic fibers were broken, and fibrous plaques were formed, and the whole vascular lumen was slightly narrowed. Otherwise, few lipid infiltration was found.

#### 3.7.4. The EOAA Group

The inner wall of the vascular was not smooth with the exfoliated ECs, and the elastic fibers were broken accompanied by the formation of atherosclerotic plaques and obvious narrowing of the whole vascular lumen, and there were more fat droplets in the plaques; it was similar to the model group.

### 3.8. The Contents of AMPK, PI3K, Akt, and eNOS mRNA in the Thoracic Aortas of Mice

The content of AMPK-mRNA in the model group was significantly lower than that in the control group (*P* < 0.05). The contents of AMPK-mRNA in the MS group, FMS group, and MFV group were significantly higher than those in the model group (*P* < 0.01, *P* < 0.05, *P* < 0.05). The content of AMPK-mRNA in the EOAA group was higher than that in the model group, but there was no significant difference (*P* > 0.05). Among the MCP groups, the content of AMPK-mRNA in the MS group was significantly higher than that in the EOAA group (*P* < 0.05), and there was no significant difference among other groups (*P* > 0.05).

The content of PI3K-mRNA in the model group was significantly lower than that in the control group (*P* < 0.01). There was no significant difference in the contents of PI3K-mRNA among the model group and the MCP groups (*P* > 0.05). The contents of PI3K-mRNA among the MCP groups had no significant difference (*P* > 0.05).

Tested by ANOVA, the contents of Akt-mRNA among all groups showed no significant difference (*P* > 0.05).

**Figure 6:**
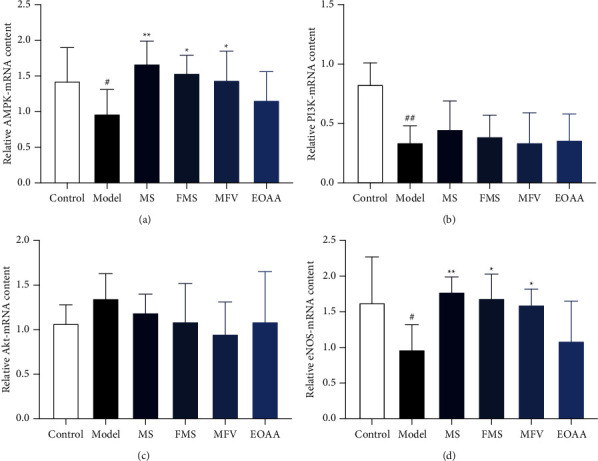
(a) The content of AMPK-mRNA. (b) The content of PI3K-mRNA. (c) The content of Akt-mRNA. (d) The content of eNOS-mRNA. Compared with the control group, ^#^*P* < 0.05, ^##^*P* < 0.01; compared with the model group, ^*∗*^*P* < 0.05, ^*∗∗*^*P* < 0.01 (mean ± SD, *n* = 12).

The content of eNOS-mRNA in the model group was significantly lower than that in the control group (*P* < 0.05). The contents of eNOS-mRNA in the MS group, FMS group, and MFV group were significantly higher than those in the model group (*P* < 0.01, *P* < 0.05, *P* < 0.05). The content of eNOS-mRNA in the EOAA group was higher than that in the model group, but there was no significant difference (*P* > 0.05). Among the MCP groups, there was no significant difference in the contents of eNOS-mRNA between the MS group and FMS group (*P* > 0.05), and there was significant difference among other groups (*P* < 0.05). These results are shown in Figures 6(a)–6(d).

## 4. Discussion

In recent years, the harmfulness of particulate matter has attracted widespread attention, which caused people to question whether the particulate matter in MS affected human health similarly. According to the study of the oxidative damage ability of PM10 [22] and PM2.5 [23] in MS, researchers did not find the phenomenon that PM10 caused oxidative damage to DNA, and the DNA oxidative damage of PM2.5 in MS was also significantly lower than that in other sources of PM2.5, so it proved the safety of particulate matter in MS. In addition, the MS can promote the alveolar type II epithelial cells (A549) and macrophages cells (NR8383) proliferation; however, when the concentration increased, it will lead to cause changes in cell viability and cell apoptosis [24, 25]. The clinical research on MS observed healthy adults who were in the MS environment with concentration of 9∼12 mg/m^3^, and the results showed that there were no significant changes in various physiological indicators, suggesting that MS was safe within a certain concentration range [26]. However, some people still felt discomfort when they were exposed to the MS environment for a period of time [27]. MS cannot be avoided in the process of burning the moxa floss, and MS was considered as one of the effective factors during moxibustion, which was proved in previous research. Therefore, how to maintain MS during moxibustion and reduce discomfort caused by MS to some people was a problem that needed to be solved urgently in the clinic.

The Whatman Cambridge filter with the particulate matter retention rate of more than 97.8% was used to filter the particulate matter in MS, and the influence of filtered particulate matter on the effect of MS was discussed [28]. The result showed that MS and FMS have no significant difference in improving lipid metabolism and vascular endothelial function. It was suggested that the particulate matter in MS has no obvious effect on anti-AS. Another research showed that when the moxibustion device was equipped with the MS barrier, it can significantly reduce the concentration of particulate matter and the mole fraction of some harmful smoke and improve the safety of moxibustion [29]. The result also provided the animal experiment support for moxibustion doctor to choose the appropriate moxibustion method in practice.

Burning moxa floss can be seen as two parts: one was in the burning part and another was in the closing burning part. Because the temperature of two parts was different, it resulted in the difference of the combustion products. The unburned moxa floss in medium and low temperature about 60°C∼150°C can be heated to produce volatile oil components, which were mainly considered as the light components in MS. MS is the main existing form of MCPs, so it can be considered that the components of MS included MFV. By comparing the effects of MS and MFV on ApoE^−/−^ mice, we wanted to explore whether the process of burning was a necessary step for moxibustion. This experiment result showed that MS was more effective than MFV in improving lipid metabolism and vascular endothelial function. Therefore, it was suggested that the burning process may be an indispensable part of moxibustion.

In this study, the existing forms of MS and EOAA were different. EOAA was directly extracted from three-year storage of *Artemisia argyi*, while MS was produced by burning the moxa floss. Secondly, the composition was different. EOAA only included the volatile oil, which belonged to the light components, while the substances of MS included heavy and light components, so the components were wider than EOAA. This study showed that MS was more effective than EOAA in improving lipid metabolism and vascular endothelial function. It was suggested that the components produced during heating, volatilization, pyrolysis, and combustion of moxa floss were the key factors for moxibustion to improve endothelium function for anti-AS.

Severe lipid metabolism disorder was the pathogenesis-based of AS, which were usually characterized by significant increase in the contents of TG, TC, LDL, and VLDL and significant decrease in the contents of HDL and other related lipoproteins. In this experiment, it was found that the contents of TC, TG, LDL-C, and ox-LDL in the model group increased significantly, while the content of ApoA-I decreased significantly. It was worth noting that the content of HDL-C increased significantly, which may be related to the genetic background of ApoE^−/−^ mice [30].The vascular bifurcation is a common site for the formation of AS plaques, especially the aortic root and aortic arch [31–33]. When 8-week-old ApoE^−/−^ mice were fed high-fat diet for 14 weeks, we found obvious atherosclerotic plaques in the thoracic aortas. After the intervention of MS, FMS, and MFV, the structure of aortic plaque and endothelial cells was improved, but there was no improvement in the EOAA group.

The AMPK-related pathways prevented and treated AS by protecting vascular function, promoting cholesterol outflow, accelerating fatty acid oxidation, and inhibiting inflammation [34]. The AMPK regulated the PI3K signal pathway in complex way and stimulated the activation of the signaling pathway. Similarly, the Akt can also feed back and regulate the phosphorylation process of AMPK [35]. There was a wide range of adjustment between PI3K/Akt and AMPK, and the relationship was mainly characterized by mutual cooperation [36]. The PI3K/Akt signal pathway played an important role in the proliferation and differentiation of vascular endothelial cells mainly through the activation of eNOS [37–39]. Some studies had shown that high-fat diet can reduce the phosphorylation of endothelial AMPK, leading to the downregulation of PI3K/Akt. The eNOS pathway was associated with endothelial dysfunction and resulted in the formation of AS finally [40]. In addition, anthocyanins can increase the content of eNOS and the production of NO by activating AMPK [41]. These results showed that the contents of AMPK and eNOS mRNA were enhanced by the intervention of MS and FMS, which further resulted in the increase of the content of NO, introducing coordination for the imbalance of NO and ET-1 and improving the function of vascular endothelial cells. It was considered that the AMPK may be related to the activation of NO signal transduction by directly affecting the activity of eNOS [42], which is worthy of further study.

We acknowledge some deficiencies in this study. First, because the dosages of EOAA were converted based on the same dosages of moxa floss, the EOAA group showed no significant effect for anti-AS, which may be the problem of insufficient dosages of EOAA. Thus, we plan to set different dosages of EOAA to screen out the optimal dosages for anti-AS further. In addition, the temperature of intervention was set at 20°C∼22°C. However, EOAA was affected by different temperatures through transdermal absorption, so different temperatures should be considered in future.

In conclusion, the clinical trial showed that moxibustion can delay the process of AS by reducing blood lipid [43], and the animal experiment also showed that the MCPs can regulate lipid metabolism [19]. According to the result of this study, MS and FMS can inhibit the progression of atherosclerosis in ApoE^−/−^ miceIt is indicated that the MCPs can regulate the lipid metabolism and reduce early lipid accumelation. Secondly, the effect of FMS was similar to MS, and it was reasonable to consider that the particulate matter in MS may not be the key factor in anti-AS. This is a value guiding for the clinical operator when they reduce the production of moxa smoke by using the smoke barrier during moxibustion, which not only ensure the effect of moxibustion, but also increase the comfort when patients received moxibustion treatment.

## Figures and Tables

**Table 1:** Primers used for RT-qPCR.

Gene marker	Primer F	Primer R
AMPK	TTGAAACCTGAAAATGTCCTGCT	GGTGAGCCACAACTTGTTCTT
PI3K	AATGTGCCCTCTTTCGTTGT	TGAATGGTGACTGGCTGACT
Akt	ACTCATTCCAGACCCACGAC	CACAATCTCCGCACCATAGA
eNOS	TGTCTGCGGCGATGTCACT	GTCTCACCCTTAGGACCAAGA
Actin	GCTCTTTTCCAGCCTTCCTT	CTTCTGCATCCTGTCAGCAA

## Data Availability

The data used to support the findings of this study are available from the corresponding author upon request.
